# Extracellular Vesicles in Prostate Cancer: New Future Clinical Strategies?

**DOI:** 10.1155/2014/561571

**Published:** 2014-02-23

**Authors:** Ilaria Giusti, Vincenza Dolo

**Affiliations:** Department of Life, Health and Environmental Sciences, University of L'Aquila, Via Vetoio-Coppito 2, I-67100 L'Aquila, Italy

## Abstract

Prostate cancer (PCa) is the most common cancer—excluding skin tumors—in men older than 50 years of age. Over time, the ability to diagnose PCa has improved considerably, mainly due to the introduction of prostate-specific antigen (PSA) in the clinical routine. However, it is important to take into account that although PSA is a highly organ-specific marker, it is not cancer-specific. This shortcoming suggests the need to find new and more specific molecular markers. Several emerging PCa biomarkers have been evaluated or are being assessed for their potential use. There is increasing interest in the prospective use of extracellular vesicles as specific markers; it is well known that the content of vesicles is dependent on their cellular origin and is strongly related to the stimulus that triggers the release of the vesicles. Consequently, the identification of a disease-specific molecule (protein, lipid or RNA) associated with vesicles could facilitate their use as novel biological markers. The present review describes several *in vitro* studies that demonstrate the role of vesicles in PCa progression and several *in vivo* studies that highlight the potential use of vesicles as PCa biomarkers.

## 1. Prostate Cancer

The prostate is an exocrine gland in the male reproductive system that is responsible for the production of seminal/prostatic fluid, a liquid that usually constitutes 50–70% of the semen volume (along with seminal vesicle fluid and, of course, spermatozoa) [1]. The mature prostate gland is composed of columnar and polarized cells lining the prostatic lumen and more elongated basal epithelial cells that separate the lumen from the stroma [2, 3]; both basal and luminal epithelial cells can mutate, thus causing prostate cancer (PCa) [2].

As with all types of cancer, PCa is the result of genetic and epigenetic alterations that induce transformations of normal glandular epithelia [4]. The dysregulation of many molecules and genes has been implicated in PCa; some of these molecules (e.g., NKX3.1, FOXA1, and Myc) seem to be relevant for cancer initiation because their expression is altered during the early stages; other pathways (*TMPRSS2-ERG* and *RB*) seem to be involved in the transition from PCa to CRPC. The PI3 K, Akt,* PTEN, *and mTOR are always dysregulated in PCa [2].

With regard to epigenetic alterations, both hypo- and hypermethylation are well documented. Hypermethylation is common in PCa and is believed to play a role in PCa initiation and progression; hypermethylation of the *GSTP1* gene promoter (which can involve the 5V region or CpG islands) is a highly specific marker for PCa, but it lacks sensitivity [5]. The dysregulation of gene expression in PCa appears to be due to changes in chromatin remodeling as well as posttranslational modifications of histones, with several histone-modifying enzymes (namely, HDACs, HMTs, and HDMs) being altered. Changes in miRNA levels are also important in PCa progression, as demonstrated by the role of miRNAs in blocking apoptosis, cell-cycle promotion, migration, invasion, and the maintenance of androgen-independent growth [6, 7].

Among nonskin cancers, PCa is the most common cancer in men older than 50 years of age [8, 9]. The etiology of PCa has not been fully elucidated; however, its risk factors are well-established and include age (incidence and mortality rates increase exponentially after 50 years of age), ethnicity (African Americans have the highest rates), and a family history of PCa (men with fathers or brothers affected by PCa have double the risk of developing this form of cancer) [8, 10, 11]. Other risk factors are likely involved, such as genetic susceptibility, and there is strong evidence from migrant studies that hormones, smoking, diet, sexual factors, and other lifestyle factors also play roles in the development of PCa [8, 10, 12, 13]; among all of these risk factors, diet seems to play a major role in the initiation, promotion, and progression of prostate cancer [13].

PCa incidence rates are generally higher in North America when compared to Western Europe, Oceania, and Asia, but the rates have increased considerably worldwide during the past half century, largely due to the advent of prostatic specific antigen (PSA) testing and its increased use, which has greatly improved diagnosis of this pathology and has highlighted an increased number of cases [14, 15]. At the same time, the extensive use of PSA screening accounts for a great reduction in the proportion of men who present with metastatic disease at the time of diagnosis and the lower mortality rate in some populations [16].

PSA is a serine protease that was first identified in 1966 in seminal fluid; in 1979, its role as tumor marker was first described. Some years later, it was approved by the U.S. Food and Drug Administration for monitoring the disease status of recurrence after definitive treatment in men with PCa, and it is now used to identify men with PCa. It seems, moreover, that PSA can even identify men who are at risk of developing PCa [17, 18]. PSA exists in the bloodstream in several specific forms, including free and complexed (e.g., *α*-1-antichymotrypsin) forms; free PSA is composed of three isoforms: i-PSA (inactive PSA), pro-PSA (proenzyme PSA), and BPH-PSA (benign prostatic hyperplasia-PSA). The measurement of total PSA and its specific forms can help to differentiate between malign (PCa) and benign conditions [17, 18].

In the blood of patients with either benign prostatic hyperplasia (BPH) or PCa, the prevailing form is the complexed form, whereas the ratio of free/total PSA is lower in PCa than in BPH. BPH-PSA and i-PSA are relatively more abundant than pro-PSA in BPH, whereas in PCa, the reverse is observed [17, 18]. It seems that higher levels of pro-PSA are associated with a higher risk for PCa in men with total PSA levels of 4–10 ng/mL [19] and with more aggressive forms of PCa, as characterized by Gleason scores ≥7 or extracapsular tumor extension [20]. The Gleason score is used to grade PCa, and it is based on the microscopic appearance of cancer tissue, which takes into account the differentiation grade of the tissue. Cancers with higher Gleason scores are more aggressive and have worse prognoses than cancers with lower scores [21].

PSA analysis in serum accompanied by digital rectal examination has been the standard method for PCa screening. Although PSA is highly organ-specific, it is not a cancer-specific marker because it cannot distinguish among indolent PCa, aggressive PCa, and benign conditions (e.g., prostate inflammation is characterized by increased levels of PSA) [18, 22]. PSA levels can even be altered by ejaculation, drugs, or prostate manipulation (particularly by catheterization or prostatic massage), thus contributing to false positives that lead to unnecessary biopsies or other clinical interventions [17, 23].

Nevertheless, in the last few years, mortality specifically attributable to PCa decreased in some countries (such as the US, Canada, Germany, Italy, Switzerland, France, and Spain). As already stated, this decrease is likely due to the introduction of PSA screening, which allows the detection of PCa at an early stage, allowing early curative treatments and improving clinical outcomes. At the same time, new surgical approaches, improved irradiation protocols, and antiandrogenic therapies likely have contributed to mortality decrease as well [24]. Antiandrogenic therapy is important because PCa depends on androgen receptor activity at all stages; standard treatment strategies for disseminated cancer are based on targeting this pathway using androgen deprivation therapy (ADT) or androgen receptor antagonists. Despite such interventions, a successful treatment effect is often followed by reactivation of the androgen receptors, leading to a recurrence of PCa (so-called “castrate-resistant PCa” or CRPC) [2].

## 2. Extracellular Vesicles

It is now widely known that cells are able to release several types of extracellular vesicles [25–28] that are not merely a form of waste elimination, as it was thought when they were discovered; instead, they act as signaling packages and are able to affect neighboring cells and the surrounding microenvironment with the messages they convey [29]. The involvement of extracellular vesicles in various physiological and pathological events, such as the immune response, cellular differentiation, and vascular and cancer pathologies, is also clear [30]. How extracellular vesicles interact with target cells remains to be fully elucidated, even if several hypotheses have been proposed—for example, direct cellular contact mediated by the interaction of membranes with target cell receptors, fusion with the plasma membrane, or encapsulation by endocytosis [26, 31].

Usually, extracellular vesicles can be isolated *in vivo* from all bodily fluids (e.g., blood, urine, semen, amniotic fluid, saliva, synovial and bronchoalveolar fluids, breast milk, spinal fluid, ascites, and malignant pleural effusion) [22, 32], particularly if they are exposed to primary tumors [33, 34].

**Figure 1:**
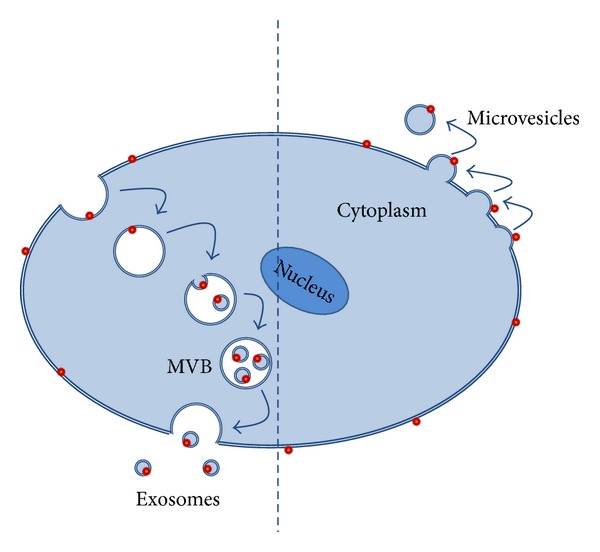
Schematic view of exosomes and microvesicles being released from a cell.

The most important extracellular vesicles released from cells are apoptotic bodies, exosomes, and shed microvesicles (MVs) (Figure 1) [25–27]. Extracellular vesicles differ mainly in their cellular origins and sizes. Apoptotic bodies are released from the cell membrane as the final consequence of cell fragmentation during apoptosis, and they have irregular shapes with a range of 1–5 *μ*m in size [26, 27]. Exosomes are released by the fusion of multivesicular bodies (MVB) with the plasma membrane and are 30–100 nm in size [26, 27]. Shed MVs are released through regulated outward budding or blebbing of the plasma membrane; they are heterogeneous in shape and are 100–1,000 nm in size [26, 27].

In addition to the well-defined differences in cell origin and size, extracellular vesicles can also exhibit differences or show overlapping features in their molecular compositions and functions. We will further discuss exosomes and MVs, which are released from viable cells and are primarily involved in cell-to-cell communication.

The membranes of exosomes are composed of several lipids, including cholesterol, ceramide, and sphingomyelin, and they are specifically characterized by low levels of phosphatidylserine exposure [26]. The contents of exosomes include mRNA, microRNA (miRNA), and several proteins (ranging from cytoskeletal proteins and adhesion molecules to proteins involved in signal transduction, transcription regulation, and antigen presentation) [35, 36].

Exosomes, secreted both *in vitro* and *in vivo, *are involved in intercellular communication in both physiological as well as pathological processes (i.e., cancer) [27, 37] with the following effects.

- Immune system modulation. Exosomes are variously involved in immune system functions and show either stimulatory or inhibitory effects, acting in antigen presentation or mediating immune tolerance [38, 39]. Exosomes constitutively released from syncytiotrophoblasts, for example, play a role in promoting fetal survival, contributing to other mechanisms that provide maternal immune tolerance of a fetus [40].
- Regulation of neuronal cell functions. Some authors have suggested that exosomes from microglia may provide an effective means of intercellular neural communication, which would be very useful considering the limited motility of such cells [41]. It has also been shown that exosomes released from oligodendroglial cells seem to be involved in the trophic support of axons and to contribute to a balanced production of proteins and lipids for myelin [42].
- Cancer progression. Tumor cells release exosomes, which can contribute to metastasis and cancer progression. Exosomes are involved in adhesion to the substratum, an important feature for metastatic cells [43], and in angiogenesis induction [44]; moreover, they contain and deliver prooncogenic miRNA to target cells [45]. Adipose tissue-derived mesenchymal stem cells treated with tumor-derived exosomes adopt a myofibroblast phenotype, and myofibroblasts are important tumor-supporting cells [46]; Fas-L-expressing exosomes induce apoptosis in T cells, playing a role in tumor immune evasion [39]. Exosomes are also involved in drug resistance: in drug-resistant human ovarian carcinoma cells, higher cisplatin export via exosomes has been observed [47].

MVs' membranes are characterized by high levels of phosphatidylserine, which is translocated from the inner to the outer surface leaflet [25], and their cargo includes proteins (e.g., enzymes, growth factors, growth factor receptors, cytokines, and chemokines), lipids, and nucleic acids (mRNA, miRNA, ncRNA, and genomic DNA) [48, 49]. Several studies of the molecular characterization of MVs have suggested that MVs are not simply miniature versions of the parental cells; instead, they show both similarities and differences with respect to the molecular composition of their cells of origin [25, 50]. For example, MVs from human gliomas contain a multitude of molecules that are not detectable or are expressed in different amounts in the parental cells from which they originate [49]. MVs have been widely studied both in normal cell types (including platelets, red blood cells, and endothelial cells) and, more frequently, in cancer cells [27, 51–53] for their well-established role in cancer progression. Indeed, MVs contribute to cancer progression in different ways [28], as described below.

- Contribution to the proinvasive characteristics of cancer cells. Tumor progression and invasion depend on the ability to modify the extracellular matrix. MVs appear to promote the proteolytic cascade required for the localized degradation of the extracellular matrix through the involvement of lytic enzymes, such as uPA, MMPs, and cathepsins, which are contained in MVs [53–57].
- Apoptosis evasion. Since MVs contain caspase 3, one of the main apoptotic enzymes, it has been suggested that tumor cells may escape apoptosis by releasing MVs enriched with caspase 3, thus preventing its intracellular accumulation [58].
- Induction of transformation. MVs derived from human cancer cells (e.g., breast carcinoma and glioma cells) are able to transform normal fibroblasts and epithelial cells to adopt typical cancer cell characteristics (e.g., anchorage-independent growth and enhanced survival capability) through the transfer of the tissue-transglutaminase enzyme [59].
- Drug resistance. Some antitumoral drugs accumulate in MVs and are expelled through them [60].
- Contribution to immunoescape. MVs mediate interactions between cancer and immune cells to modulate the immune response. MVs can carry Fas ligand, resulting in T-cell apoptosis and consequently preventing their cytotoxic effects on tumor cells [61]. The fusion of MVs from human melanomas and colorectal carcinomas with monocytes inhibits differentiation and promotes immunosuppressive cytokine release [62]. MV-associated CD46, a membrane complement inhibitor, helps cancer cells to escape from complement-induced lysis [63].
- Induction of angiogenesis. It is well known that tumor growth and survival depend on the formation of new blood vessels (i.e., angiogenesis) that infiltrate the tumor mass [64]. MV-associated EGFR can activate the VEGF/VEGFR pathway in endothelial cells [65]; MVs are a rich source of proangiogenic growth factors, such as VEGF, FGF-2, and proteases (e.g., uPA, MMPs, and cathepsin B) [52, 53, 66–69], and of the MMP stimulant EMMPRIN [70]. Lytic enzymes can favor angiogenesis and new vessel formation by carrying out degradation of the basal membrane and the extracellular matrix.

Therefore, despite clear differences in cell origins and size ranges, the specific functions and features of the different extracellular vesicle subpopulations are often overlapping and ambiguous; moreover, difficulties related to the available isolation techniques make it difficult to precisely separate the subpopulations of MVs, thereby preventing the investigation of their specific characteristics.

To further amplify the confusion about the identity of the vesicle subpopulations, in seminal/prostatic fluid, another population of vesicles called prostasomes is present [22]. Prostasomes are derived from the prostate gland and are present in high concentrations in seminal/prostatic fluid; they range in size from 50 to 500 nm (with a mean diameter of 150 nm) [22]. Prostasomes share origins with exosomes because they are stored in the MVBs of epithelial cells lining the acinar ducts of the prostate gland and are released after MVB fusion with the cell membrane [71]. Despite their overlapping origins, exosomes and prostasomes differ not only in size (as already mentioned) but also in composition: the membrane surrounding prostasomes has a specific composition with a higher concentration of cholesterol and sphingomyelin and a substantially high cholesterol/phospholipid ratio when compared to exosomes [22, 72, 73]. Furthermore, exosomes are usually characterized by a bilayer of membranes, whereas prostasomes can have a multilayer membrane [74]. Moreover, in prostasomes, chromosomal DNA, which appears to be absent in exosomes, has been reported [71, 75]. In addition to lipids and DNA, the cargo of prostasomes could also consist of proteins, such as enzymes, transport proteins, structural proteins, signal transduction proteins, and GTP-binding proteins [76].

It is not clear, however, whether prostasomes isolated *in vivo* from seminal/prostatic fluid correspond to vesicles that are isolated *in vitro*. Some authors state that in prostate secretion, only exosomes and prostasomes are present [77], whereas others have hypothesized that prostasomes are exosomes derived from prostate cells in biological conditions [22], and others consider them as belonging to the “exosome family” [71]. Like exosomes and MVs, prostasomes are involved in the exchange of information, specifically from prostate cells to other cells (in physiological conditions, recipient cells are mainly spermatozoa) [78]. Transfers of messages can involve several mechanisms, such as fusion or direct contact between the prostasome and the sperm cell membrane, initiating the internalization of prostasomes by the sperm cell [78]. Regarding their biological function, prostasomes seem to be involved mainly in human reproduction; they have a stimulatory effect on spermatozoa motility (showing a promotional effect), capacitation, and the acrosome reaction (i.e., having a regulatory role involving cholesterol transfer) as well as modulation of immunologic attacks (i.e., protecting spermatozoa from phagocytosis performed by the female's immune cells); they also show antioxidant capacities (reducing reactive oxygen species production, to which human spermatozoa are very sensitive) and antibacterial properties (inducing bacterial membrane deformation) [78–81]. In addition to their physiological role in reproduction, prostasomes also appear to be involved in PCa progression [78].

In fact, prostasomes are released not only from normal prostate cells but also from benign prostate tissue, prostate cancer cells, and poorly differentiated cells of prostate cancer metastases [81, 82]. It was suggested that prostasomes contributed to the development of PCa because they were observed in secretions from the prostate (a cancer with a very high incidence) but not in seminal vesicle secretion (which is, indeed, a cancer extremely rare) [78].

Several authors have suggested that some features of prostasomes that may have developed to sustain their physiological role in reproduction could also promote cancer cell survival and proliferation [83]. The following are several roles that have been proposed for prostasomes in cancer progression.

- Inhibition of the immune system. Prostasomes inhibit mitogen-induced proliferation in a dose-dependent manner in cytotoxic T lymphocytes; therefore, they could most likely interfere with their role (i.e., the recognition of antigens expressed on tumor cells) [84].
- Inhibition of the complement system. The complement system is involved in immune surveillance against tumor development. If cancer cells are unable to protect themselves against complement attack, they will be eliminated very early during the development of cancer. The phosphorylation of C3, a component of the complement system, results in the inhibition of both the classical and alternative pathways of complement activation [85]. PCa prostasomes are characterized by higher activities of protein kinases A and C and casein kinase II when compared to prostasomes isolated from seminal plasma. Consequently, they are able to phosphorylate the C3 component, thus inhibiting the complement [86]. Cancer prostasomes also may inhibit complement through an alternative mechanism: by expressing CD59, a glycosylphosphatidylinositol-anchored protein that prevents the full assembly of the membrane-attack complex of complements, they inhibit complement-mediated lysis [87]. Again, prostasomes from cancer cells express higher CD59 levels than those from normal cells [87].
- Induction of migration. Protein kinases A and C and casein kinase contained in prostasomes from PCa are able to perform fibrinogen phosphorylation. Fibrinogen phosphorylation inhibits fibrinolysis, making fibrinogen more resistant to cleavage and thereby more available as a substrate for cancer cells, easing their migration [78]. Additionally, prostasomes from PCa express high levels of tissue factor (TF) [88], which is known for its ability to promote cell migration [89] and is also involved in cancer progression due to its ability to induce tumor angiogenesis, cell adhesion, and invasion. Consequently, prostasome-associated TF may further contribute to tumor growth [78].
- Induction of invasion. Dipeptidyl peptidase IV is associated with prostasomes and is involved in the proteolytic cascade required for cancer progression through the ECM because the activation of plasminogen and expression of MMP-9 seem to depend on the activity of dipeptidyl peptidase IV [83].
- Induction of angiogenesis. As has already been mentioned, tissue factor is able to promote angiogenesis through FVIIa-induced VEGF expression. VEGF is a key regulator of angiogenesis due to its ability to stimulate proliferation and migration in endothelial cells [90]. Another important molecule in inducing angiogenesis is angiotensin II, which is produced from the precursor angiotensin I by the action of the angiotensin-1 converting enzyme (ACE) [91]; ACE activity is very high in seminal fluid, and it is mainly associated with the prostasomal membrane [83].

So, prostasomes, which normally have a fundamental role in physiological processes related to fertilization, might turn against the host, favoring the transition of normal cells to cancer cells. The transition from positive to negative action seems to take place at approximately 50 years of age. This is likely why a higher prevalence of PCa is observed in men over 50 years [83].

## 3. Extracellular Vesicles in Prostate Cancers

**Figure 2:**
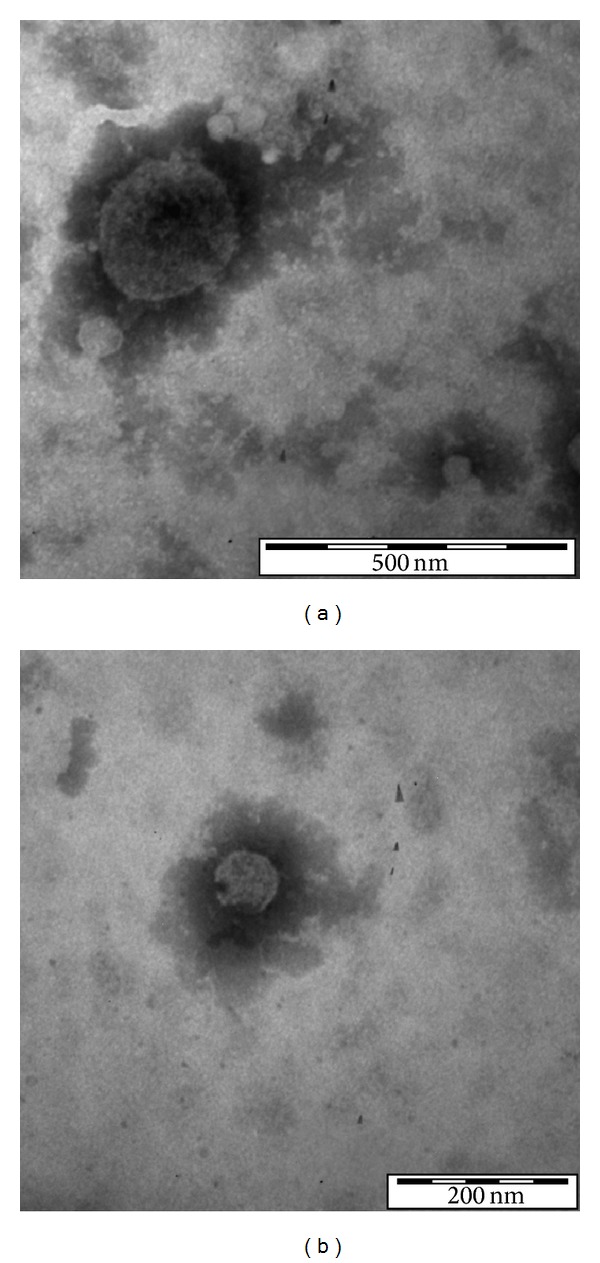
Transmission electron micrograph of extracellular vesicles released from PC3 cells. (a) Vesicle sized 248 nm (microvesicle). (b) Vesicle sized 79 nm (exosome) (personal original unpublished data).

**Figure 3:**
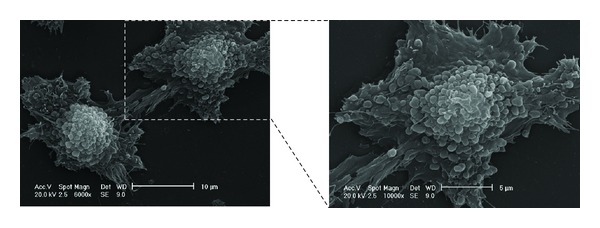
Scanning electron micrograph of PC3, a human prostate cancer cell line. Note the enormous release of microvesicles of heterogeneous dimensions ranging between 300 and 1,000 nm. Microvesicle shedding is visible over the entire cell body (personal original unpublished data).

Similarly to other cell types, PCa cells are able to release extracellular vesicles (Figures 2 and 3). Nevertheless, only a few *in vitro* studies in the literature refer to the role played by vesicles released from the PCa cells, highlighting their roles in cell-to-cell communication in cancer progression.

A deep proteomic analysis performed on vesicles from the PC3 cell line revealed that vesicles contain numerous proteins involved in the regulation of several biological processes, ranging from transport to metabolic process, response to stimuli, and cell differentiation and communication. Many were nuclear and cytosol proteins, but numerous cytoskeleton proteins were also present. Among these proteins, CDCP1 and CD151, whose involvement in PCa has already been described, stand out as possible biomarkers considering that they were more concentrated in vesicles released by prostate cancer cells than in those released by normal epithelial prostate cells [92].

PCa-derived vesicles stimulate fibroblast activation, a fundamental requirement for the induction of a favorable niche for cancer development, by increasing their motility and protecting them from apoptosis—events that are partially due to an increase in ERK1/2 phosphorylation. Vesicles from fibroblasts thus activated are, in turn, able to induce migration and invasion in the PC3 cell line, supporting cancer pathogenicity. It seems that the chemokine receptor CX3CR1 also plays a role in this process [93].

PCa tissue releases MVs capable of degrading collagen IV and the reconstituted basal membrane Matrigel. MVs released from PC3 cells (a highly metastatic PCa cell line) have been found to enhance the adhesive and invasive capabilities of LnCaP (a poorly invasive PCa cell line) [67].

It was also suggested that vesicles from hormone-refractory PCa cells are able to induce mouse osteoblast differentiation via Ets1 contained in them, suggesting a role for vesicles in cell-to-cell communication during the osteoblastic metastasis process [94]. It is well known that in osteoblastic metastasis, a vicious circle develops between PCa cells and osteoblasts/osteoclasts, with PCa cells providing growth factors to osteoblasts and osteolytic factors (e.g., BMPs, TGF-*β*, IGF, FGF, PDGF, ET1, VEGF, and MMPs) that activate these cells and allow them to produce bone-derived growth factors (e.g., PDGF, BMPs, TGF*β*, IGFs, and FGFs) that further induce cancer-cell stimulation [94, 95]. Moreover, the release of vesicles from PCa cells is induced from osteoblast-conditioned media, further suggesting that vesicles contribute to communication between cells in the course of this vicious circle [96].

A recent study highlights the role of vesicles in tumor microenvironment cell-to-cell communication, showing that vesicles released from the human prostate carcinoma cell line DU145 are able to induce transformation in the nonmalignant human prostate epithelial cell line, as evidenced by anchorage-independent growth in soft agar which is a typical feature of malignant cells. Additionally, vesicles isolated from PCa patients with a Gleason grade of 2 have similarly been used to treat nonmalignant prostate cells and have induced soft agar colony formation. The same study, however, suggested that vesicles could potentially be used to reverse the cancer phenotype because vesicles isolated from nonmalignant cells inhibited the growth of carcinoma cells in soft agar.

PCa vesicles are also involved in drug resistance: DU145 cells, which are normally sensitive to camptothecin treatment, became resistant to camptothecin-induced apoptosis after being cocultured with vesicles isolated from the camptothecin-resistant cell line RC1. Conversely, RC1 cells, cocultured with vesicles isolated from DU145, underwent apoptosis when treated with camptothecin, suggesting the role of vesicles in mediating drug resistance. Several molecules seem to be involved in these processes, including SOCS3 and STAT3 [97].

In addition to the *in vitro* studies that have tried to understand the roles played by vesicles in cancer progression, several *in vivo *studies have been performed to understand whether vesicle number or some vesicle-associated molecules could be used for diagnosis and prognosis. Although PSA is currently considered the gold standard for the detection of PCa, it is important to take into account not only PSA's high organ specificity but also its lack of cancer specificity. PSA also gives no indication about the proliferation and metastatic potential of prostate cancer cells [78], hence the need to find new and more specific molecular markers to assist or replace PSA.

To this end, PCa was studied indirectly by analyzing biological fluids in a search for useful protein, DNA, and RNA markers [5]. Several emerging biomarkers have been evaluated or are being assessed for their potential use. Biomarker research looks at all useful specimens, as identified below.

- Prostate tissue. Tissues from PCa have been studied not only to understand PCa pathophysiology but also to find new biomarkers. The reliability of this strategy, however, is based on the correct tissue sampling: if the biopsy misses the tumor, even an optimal biomarker will fail to detect cancer [98]. Several molecules from prostate tissue are possible candidates, such as Ki-67, p53, Bcl-2, AMACR, PSMA, BMP-6, PTEN, NF-*κ*B, CYCS, ICK, IKBKB, GAD1, CD10, and syndecan-1 [5].
- Blood. Several useful techniques, ranging from ELISA to capillary electrophoresis coupled to mass spectrometry, are being studied to facilitate the accurate evaluation of plausible markers. Such studies have shown, however, that blood may present some technical problems in tissue sampling that may make it an unreliable source of biomarkers—this point follows from the fact that the amount of proteins being studied may depend on several factors (such as clotting time). Consequently, the use of serum or plasma may be more advisable [98]. Some blood markers are EPCA and PSCA; serum markers include Crisp-3, hK2, OPG, CGA, TGF-*β*, hK2, IL-6, Cav-1, E-cadherin, EGFR, VEGF, von Willebrand factor, alpha-1 chymotrypsin, vilin, hepsin, neuron-specific enolase, *β*-catenin, and hK11 [5].
- Urine. The use of urine for sampling has several obvious advantages. Samples are abundant, they are obtainable in a noninvasive way, and they have greater stability than tissue or blood samples; moreover, there are no difficulties related to sampling. Because urine can contain both exfoliated PCa cells and PCa-secreted products, it could be considered a potential source of markers for early detection [98]. Useful markers are TMPRSS2-ERG oncogenic gene fusion rearrangement, PCA3/DD3, Survivin, telomerase, Tb15, Bradeion, MCM-5, hepsin, *δ*-catenin, Lgals3, CFB, Apo-D, RECK, PECAM1, and others [5, 23, 99].
- Seminal plasma. As with urine, seminal plasma has the advantage of being easily accessible and highly stable. Several biomarker candidates (N-acetyllactosaminide beta-1,3-N-acetylglucosaminyltransferase, prostatic acid phosphatase, stabilin-2, GTPase IMAP family member 6, and semenogelins-1 and -2) have been identified based on differential expression between PCa patients and controls [100].

The possibility of using extracellular vesicles as PCa biomarkers has generated considerable interest. Because the contents of these vesicles include a tumor-enriched repertoire of biomolecules dependent on their cellular origin, strongly related to the stimulus that triggers their release, the discovery of a disease-specific protein, lipid, or RNA associated with the vesicles could make it possible to use them as novel biological markers for prognostic and diagnostic purpose and for monitoring of the disease, not only in PCa but virtually in all types of cancers [22, 50]; indeed, several studies in this direction have been already conducted in several cancer diseases (Table 1). The findings that urine from cancer patient is characterized by elevated exosome secretion [101] and that prostasomes can be detected at higher levels in plasma from PCa patients if compared to patients with nonmalignant prostate pathologies or indolent PCa [71] further support this hypothesis. For the moment, the focus is primarily on vesicle-associated miRNA, which shows great potential in urologic cancers under diagnostic and prognostic profile [102].

miRNAs are short (19–26 nucleotides long), single-stranded, noncoding RNAs that are responsible for the regulation of gene expression at the posttranscriptional level because they inhibit mRNA translation at the initiation or elongation step, thereby blocking the translation of mRNAs into corresponding proteins [109]. Changes in miRNA expression mainly affect cell proliferation, apoptosis, differentiation, and cell-cycle regulation, thus explaining the role that miRNA plays in tumor cell survival and growth, which are undoubtedly involved in cancer development and progression [110, 111]. The consequences of changes in miRNA levels include the altered expression of target oncogenes and tumor suppressor genes. Indeed, it has been widely shown that a substantial number of miRNAs that normally act as tumor suppressors are downregulated in cancer cells, whereas miRNAs normally acting as oncogenes are expressed at higher levels in cancer cells [32, 112]. It was also demonstrated *in vitro* that associated exosomal miRNAs can downregulate their target genes in recipient cells [113].

Over the years, several miRNAs have been studied for their biological role in PCa. For example, miR-20a and miR-125b are oncogenic miRNAs that have antiapoptotic and pro-survival effects, respectively, in PCa cells. miR-221 and miR-222 contribute to cancer growth; miR-126 acts as tumor suppressor, and its loss could contribute to PCa progression. Additionally, miR-146a is a tumor suppressor [111].

Because miRNAs are attractive as potential diagnostic/prognostic PCa biomarkers and may potentially be used to monitor treatment response, their levels and profiles have been studied in PCa tissue and compared with healthy tissue [32, 102, 114]. Fifteen miRNAs have been observed to be differentially expressed in PCa and benign tissue and demonstrate up to 84% accuracy for discrimination between these categories; 10 of these 15 miRNAs (namely, miR-16, miR-31, miR-125b, miR-145, miR-149, miR-181b, miR-184, miR-205, miR-221, and miR-222) exhibit downregulation, whereas the remaining 5 (namely, miR-96, miR-182, miR-182∗, miR-183, and miR-375) exhibit upregulation. miR-96 expression, moreover, has been associated with cancer recurrence after radical prostatectomy [115].

It is important to keep in mind that miRNAs are not only present within cells but they can also be released *in vitro* into cell culture media. Moreover, miRNAs have been identified *in vivo* in several biological fluids, such as blood, urine, breast milk, and seminal plasma [32, 116]. Although controversy has existed over whether miRNAs circulate freely or are encapsulated in vesicles, some studies have demonstrated that in biological fluids (specifically saliva and urine), the concentration of miRNAs was consistently higher in vesicles (especially exosomes) compared to the vesicle-depleted supernatant [117]. miRNAs can also be contained in apoptotic bodies and high-density lipoproteins or associated with Ago1 and Ago2 proteins; all of these forms of association are likely responsible for protecting the molecules from degradation secondary to RNase treatment [32, 102].

A large number of studies have evaluated the presence of miRNAs in serum or plasma, assessing the differences between PCa patients and healthy controls [32]. miR-375 and miR-141 have been demonstrated to be the most consistently associated with the pathological stage and Gleason score [102, 116]; their levels are higher in the serum of patients with castration-resistant prostate cancer than in the serum of low-risk, localized patients [118]. Additionally, miR-141 levels have been able to distinguish patients with prostate cancer from healthy controls [119].

Some of these studies specifically considered miRNA associated with serum-derived vesicles. Twelve miRNAs were differentially expressed in prostate cancer patients compared with controls, and the levels of 11 miRNAs were significantly increased in PCa patients with metastases compared to patients without metastases (vesicle-associated miRNA-141 and miRNA-375 were confirmed to be associated with metastatic PCa), suggesting that circulating miRNAs could be used to diagnose and stage prostate cancer [120].

Using exosomes as biomarkers contained in urine would be even more preferable. Because urine passes through the prostate before being discharged, miRNA features in urine would reflect the status of tumor cells [102]. When compared to blood, urine offers several advantages. Particularly, the samples can be obtained in a noninvasive way and in large quantities; moreover, the composition of urine is undoubtedly less complex than that of blood, leading to easier sample analyses [32].

Apart from studies on exosome-associated miRNAs, the exosomal content from PCa patient samples has been variably evaluated.

The amount of urinary exosomes decreases after androgen deprivation therapy, and some PCa markers (specifically, PSA, PSMA, and tumor-associated marker T54) have sometimes been detected in urinary exosomes but never in healthy donor samples; in one patient, the decrease of exosomal PSA was clearly related to treatment [101]. Even if the authors admit that the future of urine-exosome analysis in PCa remains uncertain, the use of urinary exosomes could eventually be a noninvasive approach that provides clinically useful information.

RNA expression analyses in urine-derived exosomes from patients with PCa have further confirmed the possibility of using such vesicles for new methods of diagnosis. For example, exosomes from patients with high PSA levels and high Gleason scores expressed the mRNA transcript for the fusion gene TMPRSS2:ERG, whereas PCA3 was detectable in exosomes from all patients (mRNA of TMPRSS2:ERG and PCA3 being PCa biomarkers). TMPRSS2:ERG and PCA3 were not detectable in patients treated with ADT nor in medically castrated or prostatectomized patients with verified bone metastases [77]. The Nilsson study established the potential use of urine-derived exosomal mRNA to obtain information on tumor status. The presence of tumor-specific transcripts in vesicles, moreover, is not limited to PCa but it is also present in other cancers suggesting that tumor-specific transcripts contained in vesicles could serve for diagnostic of cancer diseases; in Ewing's sarcoma (ES), for example, it has been found that both exosomes and microvesicles contained the ES specific transcript EWS-FLI1, which is not present in healthy donors and might be useful as a noninvasive diagnostic ES marker in peripheral blood [121, 122].

The circulating levels of survivin (a member of the “inhibitor of apoptosis” family), either free or contained in serum/plasma-derived exosomes, have been found to be lower in patients with benign prostatic hyperplasia and in healthy controls when compared to PCa patients. Circulating survivin levels remain high both in subjects with low and high Gleason scores, suggesting that it may be useful as a biomarker even for the earlier detection of PCa [123].

Some studies on prostasomes have shown that they could be used as potential PCa biomarkers. Using a method called 4PLA, which is a variant of the proximity ligand assay and has high sensitivity and specificity for prostasomes, the researchers demonstrated that plasma from PCa patients contains high levels of prostasomes. Moreover, the assay seemed to be able to discriminate between patients with medium and high Gleason score from those with low Gleason score. The authors suggested that the loss of prostate epithelial cell polarity, typical of PCa, could be involved in the modification of prostasome features. This method could potentially be used for early diagnosis or for monitoring responses to treatment [34].

## 4. Conclusions

Over the years, many studies have been conducted to better understand the role of extracellular vesicles circulating in biological fluids in various clinical tumor conditions and their potential use as biomarkers for prognostic or diagnostic purpose or as vaccine to induce immune response [124, 125]; some clinical studies have been already conducted to evaluate the use of vesicles in this form of immunotherapy (Table 2). The other forms of clinical application of extracellular vesicles need, instead, a further evaluation. Thus, a deeper understanding of the roles of extracellular vesicles in cell-to-cell communication and in prostate cancer biology, as well as continued expansion of the field of vesicle research, may lead to the development of extremely useful vesicular biomarkers for determining the diagnosis or prognosis of cancer. Such biomarkers may serve as valid instruments with which to assess the responses to clinical treatments. However, many questions remain about the effective role of extracellular vesicles in prostate physiological and pathological processes, and further studies are needed to clarify their usefulness as biomarkers. Furthermore, it is necessary to refine the techniques used to isolate and quantify, in blood or other biological fluids, vesicles specifically derived from tumor tissues as well as to standardize sample collection and analytical methodologies.

## Figures and Tables

**Table 1:** Summary of some studies in which tumor EVs have been assessed for their potential clinical use in disease monitoring and diagnosis of cancer patients.

Cancer type	Evidences reported in the paper	Reference
Ovarian cancer	32 of 63 plasma samples from ovarian cancer patients contained exosomes containing claudin-4, a protein that is frequently overexpressed in ovarian cancers. Only 1 of 50 samples from control patients, instead, contained claudin-4-positive exosomes. The assay of exosomes-associated claudin-4 in blood could be useful, alone or in combination with other screening methods, for the detection of ovarian cancer.	[103]

Ovarian cancer	Exosomes purified from plasma of patients with ovarian cancer carried cancer-specific miRNAs; women with early or advanced cancer showed similar miRNAs profiles, whereas healthy women or patients with benign ovarian disease expressed very different profiles. Thus, miRNA profiles of circulating exosomes could be used as diagnostic marker.	[104]

Glioblastoma	Mutant mRNAs and miRNAs specific for gliomas can be detected in microvesicles from serum of glioblastoma patients. In 7 of 25 samples, for example, EGFRvIII was detected (the tumor-specific mutant splice variant of EGFR mRNA typical of many gliomas), but it was not found in serum exosomes from 30 control patients. Moreover, levels of miRNA-21, usually overexpressed in glioblastoma tumors, were higher in serum microvesicles from glioblastoma patients than in control patients. So, tumor-derived microvesicles could be used to obtain diagnostic information.	[49]

Bladder cancer	This pilot study showed that microvesicles from urine of cancer patients contained 8 proteins whose levels were elevated, suggesting that protein composition of microvesicles could be used in early detection of bladder cancer.	[105]

Gastric cancer	Platelet microparticles plasma levels were assessed in patient with gastric cancer. Levels were significantly higher in the patients than in the healthy controls and higher in patients with stage IV disease than those in patients with lower stages (I/II/III). Plasma levels of platelet microparticles had a high diagnostic accuracy and might be useful to identify metastatic gastric patients.	[106]

Mucinous adenocarcinomas	Microparticles from blood of patients with breast and pancreatic cancer had significantly increased levels of tissue factor (TF) compared with healthy controls. Patients with higher levels of TF and MUC1 (epithelial mucin) in MVs were associated with a lower survival rate at 3–9 month followup compared to those with low TF-activity and no MUC1 expression, suggesting the possible use of plasma vesicles in prognosis of disease.	[107]

Hormone refractory prostate cancer	In patients with hormone-refractory prostate cancer, platelet MVs levels were predictive of outcome; overall survival was significantly shorter in those patients with MVs level above the cut-off compared to those patients whose level was below it.	[108]

**Table 2:** Summary of clinical trials that assessed or are evaluating the application of EVs in anticancer therapy.

Cancer type	Phase of study	State	Purpose of clinical trials and outcome	References
Non-small-cell lung carcinoma (NSCLC)	Phase I	USA	The study intended to use exosomes carrying specific antigens to activate immune response against established tumours. Exosomes from dendritic cells (DCs) obtained through leukophoresis were collected from patients with advanced NSCLC with tumor expression of MAGE-A3 or -A4 antigens. These exosomes, loaded with specific MAGE peptides, were administrated to patients to induce immune response. This form of immunotherapy was well tolerated; in 3 of 9 patients, who had no reactivity to MAGE before immunization, an increased systemic immune response against MAGE, an increased NK cells lytic activity, and a long term stabilization of disease in some patients were observed.	[126]

Melanoma	Phase I	France	The study was intended to asses a DCs-derived exosomes based vaccination in melanoma patients; autologous exosomes pulsed with MAGE 3 peptides were used to induce the immunization in patients with melanoma at stages III and IV. The study confirmed the feasibility of exosomes production in large scale, the safety of their administration to patients, and the good tolerance in cancer patients; nevertheless, even if treatment induced minor or partial responses in some patients, no MAGE3 specific T-cell immune responses were detected in peripheral blood of the same patients.	[127]

Colorectal cancer	Phase I	China	The study wanted to assess the possibility to use exosomes in immunotherapy and reported that exosomes derived from ascites, if subcutaneously administrated with GM-CFS (granulocyte macrophage colony-stimulating factor) in patients with colorectal cancer, were able to induce an antigen-specific anticancer cytotoxic T lymphocyte response. Toxicity of exosomes was minimal and patients tolerated very well the administration.	[128]

NSCLC	Ongoing phase II	France	The study aims to assess the efficacy of a therapeutic vaccine constituted by autologous DC-derived exosomes in nonoperable and advanced NSCLC patients (stages IIIB and IV), to verify if they are able to stimulate the patients' natural defenses in order to obtain the stop of tumor progression or tumor regression.	[129]

## Abbreviations

ACE: Angiotensin-1 converting enzyme
ADT: Androgen deprivation therapy
Akt: Protein kinase B
AMACR: Alpha-methylacyl CoA racemase
Apo-D: Apolipoprotein D
Bcl-2: B-cell lymphoma 2
BMP: Bone morphogenetic protein
BPH: Benign prostatic hyperplasia
Cav-1: Caveolin 1
CFB: Complement factor B
CGA: Chromogranin A
Crisp-3: Cysteine-rich secretory protein 3
CRPC: Castrate-resistant PCa
CX3CR1: Chemokine (C-X3-C motif) receptor 1
CYCS: Cytochrome c, somatic
DCs: Dendritic cells
EGFR: Epithelial growth factor receptor
EMMPRIN: Extracellular matrix metalloproteinase inducer
EPCA: Early prostate cancer antigen
ERK-1: Extracellular regulated kinase 1
ES: Ewing's sarcoma
ET1: Endothelin 1
EWS/FLI1: Ewing's sarcoma/Friend leukemia integration 1
FGF: Fibroblast growth factor
GAD1: Glutamate decarboxylase 1
GM-CFS: Granulocyte macrophage colony-stimulating factor
GSTP1: Glutathione S-transferase p1
HDACs: Histone deacetylases
HDM: Histone demethylases
hK11: Human tissue kallikrein 11
hK2: Human kallikrein 2
HMTs: Histone methyltransferases
ICK: Intestinal cell kinase
IGF: Insulin-like growth factor
IKBKB:*κ*: Inhibitor of NF-B kinase subunit beta
IL-6: Interleukin 6
LGALS3: Lectin, galactoside-binding, soluble, 3
MAGE: Melanoma-associated antigen
MCM-5: Mini-chromosome maintenance 5
miRNA: Micro-RNA
MMPs: Matrix metalloproteinases
mTOR: Mammalian target of rapamycin
MVB: Multivesicular bodies
MVs: Microvesicles
ncRNA: Noncoding RNA
NF-*κ*B:*κ*: Nuclear factor-B
NK: Natural killers
NSCLC: Non-small-cell lung carcinoma
OPG: Osteoprotegerin
PCa: Prostate cancer
PCA3/DD3: Prostate cancer antigen 3/differential display code 3
PDGF: Platelet-derived growth factor
PECAM1: Platelet endothelial cell adhesion molecule
PI3 K: Phosphatidylinositide 3-kinases
PSA: Prostate-specific antigen
PSCA: Prostate stem cell antigen
PSMA: Prostate-specific membrane antigen
PTEN: Phosphatase and tensin homolog
RB: Retinoblastoma
RECK: Reversion-inducing-cysteine-rich protein with kazal motifs
SOCS3: Suppressor of cytokine signaling 3
STAT3: Signal transducer and activator of transcription 3
Tb15: Thymosin beta 15
TF: Tissue factor
TGF-*β*: Transforming growth factor-beta
TMPRSS2-ERG: Transmembrane protease serine 2/ETS-related gene
uPA: Urokinase-type plasminogen activator
VEGF: Vascular endothelial growth factor.

## References

[1] Huggins C, Scott WW, Heinen JH (1942). Chemical composition of human semen and of the secretions of the prostate and seminal vehicles. *American Journal of Physiology*.

[2] Schrecengost R, Knudsen KE (2013). Molecular pathogenesis and progression of prostate cancer. *Seminars in Oncology*.

[3] Nelson WG, De Marzo AM, Isaacs WB (2003). Prostate cancer. *The New England Journal of Medicine*.

[4] Konishi N, Shimada K, Ishida E, Nakamura M (2005). Molecular pathology of prostate cancer. *Pathology International*.

[5] Bhavsar T, McCue P, Birbe R (2013). Molecular diagnosis of prostate cancer: are we up to age?. *Seminars in Oncology*.

[6] Coppola V, De Maria R, Bonci D (2010). MicroRNAs and prostate cancer. *Endocrine-Related Cancer*.

[7] Jerónimo C, Bastian PJ, Bjartell A (2011). Epigenetics in prostate cancer: biologic and clinical relevance. *European Urology*.

[8] Hsing AW, Chokkalingam AP (2006). Prostate cancer epidemiology. *Frontiers in Bioscience*.

[9] Gomella LG, Johannes J, Trabulsi EJ (2009). Current prostate cancer treatments: effect on quality of life. *Urology*.

[10] Hsing AW, Devesa SS (2001). Trends and patterns of prostate cancer: what do they suggest?. *Epidemiologic Reviews*.

[11] Crawford ED (2003). Epidemiology of prostate cancer. *Urology*.

[12] Hemminki K, Rawal R, Bermejo JL (2005). Prostate cancer screening, changing age-specific incidence trends and implications on familial risk. *International Journal of Cancer*.

[13] Wolk A (2005). Diet, lifestyle and risk of prostate cancer. *Acta Oncologica*.

[14] Potosky AL, Miller BA, Albertsen PC, Kramer BS (1995). The role of increasing detection in the rising incidence of prostate cancer. *Journal of the American Medical Association*.

[15] McDavid K, Lee J, Fulton JP, Tonita J, Thompson TD (2004). Prostate cancer incidence and mortality rates and trends in the United States and Canada. *Public Health Reports*.

[16] Jemal A, Ward E, Wu X, Martin HJ, McLaughlin CC, Thun MJ (2005). Geographic patterns of prostate cancer mortality and variations in access to medical care in the United States. *Cancer Epidemiology Biomarkers and Prevention*.

[17] Loeb S, Catalona WJ (2008). What to do with an abnormal PSA test. *Oncologist*.

[18] Pienta KJ (2009). Critical appraisal of prostate-specific antigen in prostate cancer screening: 20 years later. *Urology*.

[19] Khan MA, Partin AW, Rittenhouse HG (2003). Evaluation of proprostate specific antigen for early detection of prostate cancer in men with a total prostate specific antigen range of 4.0 To 10.0 ng/ml. *Journal of Urology*.

[20] Catalona WJ, Bartsch G, Rittenhouse HG (2004). Serum pro-prostate specific antigen preferentially detects aggressive prostate cancers in men with 2 to 4 ng/ml prostate specific antigen. *Journal of Urology*.

[21] Brimo F, Montironi R, Egevad L (2013). Contemporary grading for prostate cancer: implications for patient care. *European Urology*.

[22] Duijvesz D, Luider T, Bangma CH, Jenster G (2011). Exosomes as biomarker treasure chests for prostate cancer. *European Urology*.

[23] Lu Q, Zhang J, Allison R (2009). Identification of extracellular *δ*-catenin accumulation for prostate cancer detection. *Prostate*.

[24] Bouchardy C, Fioretta G, Rapiti E (2008). Recent trends in prostate cancer mortality show a continuous decrease in several countries. *International Journal of Cancer*.

[25] Rak J (2010). Microparticles in cancer. *Seminars in Thrombosis and Hemostasis*.

[26] Mathivanan S, Ji H, Simpson RJ (2010). Exosomes: extracellular organelles important in intercellular communication. *Journal of Proteomics*.

[27] György B, Módos K, Pállinger É (2011). Detection and isolation of cell-derived microparticles are compromised by protein complexes resulting from shared biophysical parameters. *Blood*.

[28] Giusti I, D’Ascenzo S, Dolo V (2013). Microvesicles as potential ovarian cancer biomarkers. *Biomed Research International*.

[29] Schifferli JA (2011). Microvesicles are messengers. *Seminars in Immunopathology*.

[30] Enjeti AK, Lincz LF, Seldon M (2008). Microparticles in health and disease. *Seminars in Thrombosis and Hemostasis*.

[31] Théry C, Ostrowski M, Segura E (2009). Membrane vesicles as conveyors of immune responses. *Nature Reviews Immunology*.

[32] Hessvik NP, Sandvig K, Llorente A (2013). Exosomal miRNAs as biomarkers for prostate cancer. *Frontiers in Genetics*.

[33] Rabinowits G, Gerçel-Taylor C, Day JM, Taylor DD, Kloecker GH (2009). Exosomal microRNA: a diagnostic marker for lung cancer. *Clinical Lung Cancer*.

[34] Tavoosidana G, Ronquist G, Darmanis S (2011). Multiple recognition assay reveals prostasomes as promising plasma biomarkers for prostate cancer. *Proceedings of the National Academy of Sciences of the United States of America*.

[35] Al-Nedawi K, Meehan B, Micallef J (2008). Intercellular transfer of the oncogenic receptor EGFRvIII by microvesicles derived from tumour cells. *Nature Cell Biology*.

[36] Valadi H, Ekström K, Bossios A, Sjöstrand M, Lee JJ, Lötvall JO (2007). Exosome-mediated transfer of mRNAs and microRNAs is a novel mechanism of genetic exchange between cells. *Nature Cell Biology*.

[37] Vlassov AV, Magdaleno S, Setterquist R, Conrad R (2012). Exosomes: current knowledge of their composition, biological functions, and diagnostic and therapeuticpotentials. *Biochimica and Biophysica Acta*.

[38] Tan A, Rajadas J, Seifalian AM (2013). Exosomes as a nannotheranostic delivery platforms for gene therapy. *Advanced Drug Delivery Reviews*.

[39] Abusamra AJ, Zhong Z, Zheng X (2005). Tumor exosomes expressing Fas ligand mediate CD8^+^ T-cell apoptosis. *Blood Cells, Molecules, and Diseases*.

[40] Mincheva-Nilsson L, Baranov V (2010). The role of placental exosomes in reproduction. *American Journal of Reproductive Immunology*.

[41] Potolicchio I, Carven GJ, Xu X (2005). Proteomic analysis of microglia-derived exosomes: metabolic role of the aminopeptidase CD13 in neuropeptide catabolism. *Journal of Immunology*.

[42] Krämer-Albers E-M, Bretz N, Tenzer S (2007). Oligodendrocytes secrete exosomes containing major myelin and stress-protective proteins: trophic support for axons?. *Proteomics. Clinical Applications*.

[43] Koumangoye RB, Sakwe AM, Goodwin JS, Patel T, Ochieng J (2011). Detachment of breast tumor cells induces rapid secretion of exosomes which subsequently mediate cellular adhesion and spreading. *PLoS ONE*.

[44] Nazarenko I, Rana S, Baumann A (2010). Cell surface tetraspanin Tspan8 contributes to molecular pathways of exosome-induced endothelial cell activation. *Cancer Research*.

[45] Kogure T, Lin W-L, Yan IK, Braconi C, Patel T (2011). Intercellular nanovesicle-mediated microRNA transfer: a mechanism of environmental modulation of hepatocellular cancer cell growth. *Hepatology*.

[46] Cho JA, Park H, Lim EH, Lee KW (2012). Exosomes from breast cancer cells can convert adipose tissue-derived mesenchymal stem cells into myofibroblast-like cells. *International Journal of Oncology*.

[47] Safaei R, Larson BJ, Cheng TC (2005). Abnormal lysosomal trafficking and enhanced exosomal export of cisplatin in drug-resistant human ovarian carcinoma cells. *Molecular Cancer Therapeutics*.

[48] D’Souza-Schorey C, Clancy JW (2012). Tumor-derived microvesicles: shedding light on novel microenvironment modulators and prospective cancer biomarkers. *Genes & Development*.

[49] Skog J, Würdinger T, van Rijn S (2008). Glioblastoma microvesicles transport RNA and proteins that promote tumour growth and provide diagnostic biomarkers. *Nature Cell Biology*.

[50] Mause SF, Weber C (2010). Microparticles: protagonists of a novel communication network for intercellular information exchange. *Circulation Research*.

[51] Taraboletti G, D’Ascenzo S, Borsotti P, Giavazzi R, Pavan A, Dolo V (2002). Shedding of the matrix metalloproteinases MMP-2, MMP-9, and MT1-MMP as membrane vesicle-associated components by endothelial cells. *American Journal of Pathology*.

[52] Dolo V, D’Ascenzo S, Giusti I, Millimaggi D, Taraboletti G, Pavan A (2005). Shedding of membrane vesicles by tumor and endothelial cells. *Italian Journal of Anatomy and Embryology*.

[53] Giusti I, D’Ascenzo S, Millimaggi D (2008). Cathepsin B mediates the pH-dependent proinvasive activity of tumor-shed microvesicles. *Neoplasia*.

[54] DeClerck YA, Laug WE (1996). Cooperation between matrix metalloproteinases and the plasminogen activator-plasmin system in tumor progression. *Enzyme and Protein*.

[55] Ginestra A, La Placa MD, Saladino F, Cassarà D, Nagase H, Vittorelli ML (1998). The amount and proteolytic content of vesicles shed by human cancer cell lines correlates with their *in vitro* invasiveness. *Anticancer Research*.

[56] Inal JM, Ansa-Addo EA, Stratton D (2012). Microvesicles in health and disease. *Archivum Immunologiae et Therapiae Experimentalis*.

[57] Graves LE, Ariztia EV, Navari JR, Matzel HJ, Stack MS, Fishman DA (2004). Proinvasive properties of ovarian cancer ascites-derived membrane vesicles. *Cancer Research*.

[58] van Doormaal FF, Kleinjan A, Di Nisio M, Büller HR, Nieuwland R (2009). Cell-derived microvesicles and cancer. *Netherlands Journal of Medicine*.

[59] Antonyak MA, Li B, Boroughs LK (2011). Cancer cell-derived microvesicles induce transformation by transferring tissue transglutaminase and fibronectin to recipient cells. *Proceedings of the National Academy of Sciences of the United States of America*.

[60] Shedden K, Xie XT, Chandaroy P, Chang YT, Rosania GR (2003). Expulsion of small molecules in vesicles shed by cancer cells: association with gene expression and chemosensitivity profiles. *Cancer Research*.

[61] Kim JW, Wieckowski E, Taylor DD, Reichert TE, Watkins S, Whiteside TL (2005). Fas ligand-positive membranous vesicles isolated from sera of patients with oral cancer induce apoptosis of activated T lymphocytes. *Clinical Cancer Research*.

[62] Valenti R, Huber V, Iero M, Filipazzi P, Parmiani G, Rivoltini L (2007). Tumor-released microvesicles as vehicles of immunosuppression. *Cancer Research*.

[63] Whitlow MB, Klein LM (1997). Response of SCC-12F, a human squamous cell carcinoma cell line, to complement attack. *Journal of Investigative Dermatology*.

[64] Carmeliet P (2005). Angiogenesis in life, disease and medicine. *Nature*.

[65] Al-Nedawi K, Meehan B, Kerbel RS, Allison AC, Rak A (2009). Endothelial expression of autocrine VEGF upon the uptake of tumor-derived microvesicles containing oncogenic EGFR. *Proceedings of the National Academy of Sciences of the United States of America*.

[66] Dolo V, Ginestra A, Ghersi G, Nagase H, Vittorelli ML (1994). Human breast carcinoma cells cultured in the presence of serum shed membrane vesicles rich in gelatinolytic activities. *Journal of Submicroscopic Cytology and Pathology*.

[67] Angelucci A, D’Ascenzo S, Festuccia C (2000). Vesicle-associated urokinase plasminogen activator promotes invasion in prostate cancer cell lines. *Clinical and Experimental Metastasis*.

[68] Lee TH, D’Asti E, Magnus N, Al-Nedawi K, Meehan B, Rak J (2011). Microvesicles as mediators of intercellular communication in cancer—the emerging science of cellular ‘debris’. *Seminars in Immunopathology*.

[69] Taraboletti G, D’Ascenzo S, Giusti I (2006). Bioavailability of VEGF in tumor-shed vesicles depends on vesicle burst induced by acidic pH 1. *Neoplasia*.

[70] Millimaggi D, Mari M, D’Ascenzo S (2007). Tumor vesicle-associated CD147 modulates the angiogenic capability of endothelial cells. *Neoplasia*.

[71] Ronquist GK, Larsson A, Stavreus-Evers A, Ronquist G (2012). Prostasomes are heterogeneous regarding size and appearance but affiliated to one DNA-containing exosome family. *Prostate*.

[72] Arienti G, Carlini E, Polci A, Cosmi EV, Palmerini CA (1998). Fatty acid pattern of human prostasome lipid. *Archives of Biochemistry and Biophysics*.

[73] Arvidson G, Ronquist G, Wikander G, Ojteg A-C (1989). Human prostasome membranes exhibit very high cholesterol/phospholipid ratios yielding high molecular ordering. *Biochimica et Biophysica Acta*.

[74] Stridsberg M, Fabiani R, Lukinius A, Ronquist G (1996). Prostasomes are neuroendocrine-like vesicles in human semen. *Prostate*.

[75] Olsson I, Ronquist G (1990). Nucleic acid association to human prostasomes. *Archives of Andrology*.

[76] Utleg AG, Yi EC, Xie T (2003). Proteomic analysis of human prostasomes. *Prostate*.

[77] Nilsson J, Skog J, Nordstrand A (2009). Prostate cancer-derived urine exosomes: a novel approach to biomarkers for prostate cancer. *British Journal of Cancer*.

[78] Ronquist G (2012). Prostasomes are mediators of intercellular communication: from basic research to clinical implications. *Journal of Internal Medicine*.

[79] Stegmayr B, Ronquist G (1982). Promotive effect on human sperm progressive motility by prostasomes. *Urological Research*.

[80] Cross NL, Mahasreshti P (1997). Prostasome fraction of human seminal plasma prevents sperm from becoming acrosomally responsive to the agonist progesterone. *Systems Biology in Reproductive Medicine*.

[81] Sahlén G, Ahlander A, Frost A, Ronquist G, Norlén BJ, Nilsson BO (2004). Prostasomes are secreted from poorly differentiated cells of prostate cancer metastases. *Prostate*.

[82] Nilsson BO, Egevad L, Jin M, Ronquist G, Busch C (1999). Distribution of prostasomes in neoplastic epithelial prostate cells. *Prostate*.

[83] Ronquist G, Nilsson BO (2004). The Janus-faced nature of prostasomes: their pluripotency favours the normal reproductive process and malignant prostate growth. *Prostate Cancer and Prostatic Diseases*.

[84] Kelly RW, Holland P, Sibrinski G (1991). Extracellular organelles (prostasomes) are immunosuppressive components of human semen. *Clinical and Experimental Immunology*.

[85] Forsberg -. PO, Martin SC, Nilsson B, Ekman P, Nilsson UR, Engstrom L (1990). *In vitro* phosphorylation of human complement factor C3 by protein kinase A and protein kinase C. Effects on the classical and alternative pathways. *Journal of Biological Chemistry*.

[86] Babiker AA, Ronquist G, Nilsson B, Ekdahl KN (2006). Overexpression of ecto-protein kinases in prostasomes of metastatic cell origin. *Prostate*.

[87] Babiker AA, Nilsson B, Ronquist G, Carlsson L, Ekdahl KN (2005). Transfer of functional prostasomal CD59 of metastatic prostatic cancer cell origin protects cells against complement attack. *Prostate*.

[88] Fernández JA, Heeb MJ, Radtke K-P, Griffin JH (1997). Potent blood coagulant activity of human semen due to prostasome-bound tissue factor. *Biology of Reproduction*.

[89] Sato Y, Asada Y, Marutsuka K, Hatakeyama K, Sumiyoshi A (1996). Tissue factor induces migration of cultured aortic smooth muscle cells. *Thrombosis and Haemostasis*.

[90] Ollivier V, Chabbat J, Herbert JM, Hakim J, De Prost D (2000). Vascular endothelial growth factor production by fibroblasts in response to factor VIIa binding to tissue factor involves thrombin and factor Xa. *Arteriosclerosis, Thrombosis, and Vascular Biology*.

[91] Heffelfinger SC (2007). The renin angiotensin system in the regulation of angiogenesis. *Current Pharmaceutical Design*.

[92] Sandvig K, Llorente A (2012). Proteomic analysis of microvesicles released by the human prostate cancer cell line PC-3. *Molecular & Cellular Proteomics*.

[93] Castellana D, Zobairi F, Martinez MC (2009). Membrane microvesicles as actors in the establishment of a favorable prostatic tumoral niche: a role for activated fibroblasts and CX3CL1-CX3CR1 axis. *Cancer Research*.

[94] Itoh T, Ito Y, Ohtsuki Y (2012). Microvesicles released from hormone-refractory prostate cancer cells facilitate mouse pre-osteoblast differentiation. *Journal of Molecular Histology*.

[95] Clemons M, Gelmon KA, Pritchard KI, Paterson AH (2012). Bone targeted agents and skeletal-related events in breast cancer patients with bone metastases: the state of the art. *Current Oncology*.

[96] Millimaggi D, Festuccia C, Angelucci A (2006). Osteoblast-conditioned media stimulate membrane vesicle shedding in prostate cancer cells. *International Journal of Oncology*.

[97] Panagopoulos K, Cross-Knorr S, Dillard C (2013). Reversal of chemosensitivity and induction of cell malignancy of a non-malignant prostate cancer cell line upon extracellular vesicle exposure. *Molecular Cancer*.

[98] Schiffer E (2007). Biomarkers for prostate cancer. *World Journal of Urology*.

[99] Chen M, Wang K, Zhang L, Li C, Yang Y (2011). The discovery of putative urine markers for the specific detection of prostate tumor by integrative mining of public genomic profiles. *PLoS ONE*.

[100] Neuhaus J, Schiffer E, von Wilcke P (2013). Seminal plasma as a source of prostate cancer peptide biomarker candidates for detection of indolent and advanced disease. *PLoS ONE*.

[101] Mitchell PJ, Welton J, Staffurth J (2009). Can urinary exosomes act as treatment response markers in prostate cancer?. *Journal of Translational Medicine*.

[102] Huang X, Liang M, Dittmar R, Wang L (2013). Extracellular microRNAs in urologic malignancies: chances and challenges. *International Journal of Molecular Sciences*.

[103] Li J, Sherman-Baust CA, Tsai-Turton M, Bristow RE, Roden RB, Morin PJ (2009). Claudin-containing exosomes in the peripheral circulation of women with ovarian cancer. *BMC Cancer*.

[104] Taylor DD, Gercel-Taylor C (2008). MicroRNA signatures of tumor-derived exosomes as diagnostic biomarkers of ovarian cancer. *Gynecologic Oncology*.

[105] Smalley DM, Sheman NE, Nelson K, Theodorescu D (2008). Isolation and identification of potential urinary microparticle biomarkers of bladder cancer. *Journal of Proteome Research*.

[106] Kim HK, Song KS, Park YS (2003). Elevated levels of circulating platelet microparticles, VEGF, IL-6 and RANTES in patients with gastric cancer: possible role of a metastasis predictor. *European Journal of Cancer*.

[107] Tesselaar MET, Romijn FPHTM, Van Der Linden IK, Prins FA, Bertina RM, Osanto S (2007). Microparticle-associated tissue factor activity: a link between cancer and thrombosis?. *Journal of Thrombosis and Haemostasis*.

[108] Helley D, Banu E, Bouziane A (2009). Platelet microparticles: a potential predictive factor of survival in hormone-refractory prostate cancer patients treated with docetaxel-based chemotherapy. *European Urology*.

[109] Chendrimada TP, Finn KJ, Ji X (2007). MicroRNA silencing through RISC recruitment of eIF6. *Nature*.

[110] He L, Hannon GJ (2004). MicroRNAs: small RNAs with a big role in gene regulation. *Nature Reviews Genetics*.

[111] Shi X-B, Tepper CG, White RWD (2008). microRNAs and prostate cancer. *Journal of Cellular and Molecular Medicine*.

[112] Chen X, Ba Y, Ma L (2008). Characterization of microRNAs in serum: a novel class of biomarkers for diagnosis of cancer and other diseases. *Cell Research*.

[113] Montecalvo A, Larregina AT, Shufesky WJ (2012). Mechanism of transfer of functional microRNAs between mouse dendritic cells via exosomes. *Blood*.

[114] Szczyrba J, Löprich E, Wach S (2010). The microRNA profile of prostate carcinoma obtained by deep sequencing. *Molecular Cancer Research*.

[115] Schaefer A, Jung M, Mollenkopf H-J (2010). Diagnostic and prognostic implications of microRNA profiling in prostate carcinoma. *International Journal of Cancer*.

[116] Brase JC, Johannes M, Schlomm T (2011). Circulating miRNAs are correlated with tumor progression in prostate cancer. *International Journal of Cancer*.

[117] Gallo A, Tandon M, Alevizos I, Illei GG (2012). The majority of microRNAs detectable in serum and saliva is concentrated in exosomes. *PLoS ONE*.

[118] Nguyen HC, Xie W, Yang M (2013). Expression differences of circulating microRNAs in metastatic castration resistant prostate cancer and low-risk, localized prostate cancer. *Prostate*.

[119] Mitchell PS, Parkin RK, Kroh EM (2008). Circulating microRNAs as stable blood-based markers for cancer detection. *Proceedings of the National Academy of Sciences of the United States of America*.

[120] Bryant RJ, Pawlowski T, Catto JWF (2012). Changes in circulating microRNA levels associated with prostate cancer. *British Journal of Cancer*.

[121] Miller IV, Raposo G, Welsch U (2013). First identification of Ewing’s sarcoma-derived extracellular vesicles and exploration of their biological andpotential diagnostic implications. *Biology of the Cell*.

[122] Tsugita M, Yamada N, Noguchi S (2013). Ewing sarcoma cells secrete EWS/Fli-1 fusion mRNA via microvesicles. *PLoS ONE*.

[123] Khan S, Jutzy JM, Valenzuela MM (2012). Plasma-derived exosomal survivin, a plausible biomarker for early detection of prostate cancer. *PLoS ONE*.

[124] van der Pol E, Boing AN, Harrison P, Sturk A, Nieuwland R (2012). Classification, functions, and clinical relevance of extracellular vesicles. *Pharmacological Reviews*.

[125] Chaput N, Théry C (2011). Exosomes: immune properties and potential clinical implementations. *Seminars in Immunopathology*.

[126] Morse MA, Garst J, Osada T (2005). A phase I study of dexosome immunotherapy in patients with advanced non-small cell lung cancer. *Journal of Translational Medicine*.

[127] Escudier B, Dorval T, Chaput N (2005). Vaccination of metastatic melanoma patients with autologous dendritic cell (DC) derived-exosomes: results of the first phase 1 clinical trial. *Journal of Translational Medicine*.

[128] Dai S, Wei D, Wu Z (2008). Phase I clinical trial of autologous ascites-derived exosomes combined with GM-CSF for colorectal cancer. *Molecular Therapy*.

[129] Viaud S, Théry C, Ploix S (2010). Dendritic cell-derived exosomes for cancer immunotherapy: what’s next?. *Cancer Research*.

